# Supplementary material for: MetaRibo-Seq measures translation in microbiomes
Source: Nat Commun. 2020 Jun 29;11:3268. doi: 10.1038/s41467-020-17081-z (PMC7324362; doi:10.1038/s41467-020-17081-z)
Supplement: Supplementary file 10 — Supplementary Data 7 [file 41467_2020_17081_MOESM10_ESM.zip › File2/Confidence_High_Taxonomy/421135_out.krona.html]

Javascript must be enabled to view this page.

members
magnitude
magnitudeUnassigned
count
unassigned
taxon
rank

421135\_out

14

2
superkingdom
14

14
phylum
1224

1236
class
14

14
order
91347


SRS057478\_contig\_number\_contig-100\_5844.49861SRS097889\_contig\_number\_20013
14
2
543
family

561
genus
12


SRS012273\_contig\_number\_27529SRS017191\_contig\_number\_16723SRS019068\_contig\_number\_28324SRS046502\_contig\_number\_contig-100\_868.869SRS049402\_contig\_number\_17371SRS064276\_contig\_number\_contig-100\_2338.154936SRS076876\_contig\_number\_contig-100\_3260.3261SRS077127\_contig\_number\_1140SRS140492\_contig\_number\_contig-100\_6414.148888SRS144362\_contig\_number\_43136SRS146888\_contig\_number\_11686SRS147022\_contig\_number\_14717
12
species
562
